# Surgical treatment of infective endocarditis in active intravenous drug users: a justified procedure?

**DOI:** 10.1186/1749-8090-9-58

**Published:** 2014-03-24

**Authors:** Alexander Weymann, Tobias Borst, Aron-Frederik Popov, Anton Sabashnikov, Christopher Bowles, Bastian Schmack, Gabor Veres, Nicole Chaimow, Andre Rüdiger Simon, Matthias Karck, Gábor Szabo

**Affiliations:** 1 Department of Cardiac Surgery, Heart and Marfan Center, University Hospital of Heidelberg, Im Neuenheimer Feld 110, Heidelberg 69120, Germany; 2 Department of Cardiothoracic Transplantation & Mechanical Circulatory Support, Royal Brompton and Harefield NHS Foundation Trust, Hill End Road, Harefield, Middlesex, London UB9 6JH, UK; 3 Pharmacy Department, University Hospital of Heidelberg, INF 670, Heidelberg 69120, Germany; 4 Department of Thoracic and Cardiovascular Surgery, University Hospital Göttingen, Robert-Koch-Straße 40, Göttingen 37075, Germany

**Keywords:** Infective endocarditis, Intravenous drug abuse, Cardiac surgery, Heart valve

## Abstract

**Background:**

Infective endocarditis is a life threatening complication of intravenous drug abuse, which continues to be a major burden with inadequately characterised long-term outcomes. We reviewed our institutional experience of surgical treatment of infective endocarditis in active intravenous drug abusers with the aim of identifying the determinants long-term outcome of this distinct subgroup of infective endocarditis patients.

**Methods:**

A total of 451 patients underwent surgery for infective endocarditis between January 1993 and July 2013 at the University Hospital of Heidelberg. Of these patients, 20 (7 female, mean age 35 ± 7.7 years) underwent surgery for infective endocarditis with a history of active intravenous drug abuse. Mean follow-up was 2504 ± 1842 days.

**Results:**

Staphylococcus aureus was the most common pathogen detected in preoperative blood cultures. Two patients (10%) died before postoperative day 30. Survival at 1, 5 and 10 years was 90%, 85% and 85%, respectively. Freedom from reoperation was 100%. Higher NYHA functional class, higher EuroSCORE II, HIV infection, longer operating time, postoperative fever and higher requirement for red blood cell transfusion were associated with 90-day mortality.

**Conclusions:**

In active intravenous drug abusers, surgical treatment for infective endocarditis should be performed as extensively as possible and be followed by an aggressive postoperative antibiotic therapy to avoid high mortality. Early surgical intervention is advisable in patients with precipitous cardiac deterioration and under conditions of staphylococcal endocarditis. However, larger studies are necessary to confirm our preliminary results.

## Background

Infective endocarditis (IE) is a serious clinical condition with an annual incidence in the general population of approximately 1.7 - 6.2 cases per 100,000 patients with a mortality of 20-30% [1]. However, the incidence in intravenous drug users (IVDUs) is 2% to 5% and is responsible for 5% to 10% of deaths in this exceptional patient cohort [2]. IE in IVDUs differs substantially from that typically observed in the general population in terms of microbiology, the involvement of multiple heart valves and prognosis [3].

The diagnosis of IE is challenging and may lead to increased mortality if the characteristic oslerian manifestations are absent, particularly among IVDU patients infected with with S. aureus and HACEK (Haemophilus species, Actinobacillus actinomycetemcomitans, Cardiobacterium hominis, Eikenella corrodens, Kingella kingae) [4]. Polymicrobial endocarditis is a variant of IE, which often occurs in IVDUs with a fatal outcome, especially when Candida species, Bartonella spp. or Tropheryma whipplei are involved [5]. Standard antibiotics are frequently not particularly effective because these microorganisms are attached in a protective biofilm form to cardiac structures, thereby rendering them inaccessible to antibiotics [3]. If valve destruction progresses and the infectious constellation is no longer controllable, early surgical treatment becomes mandatory. The surgeon generally faces a very sick patient, often suffering from severe sepsis and congestive heart failure. In this situation, the surgical rule not to implant prosthetic material into infected tissue unavoidably has to be broken and balanced against the risk of early reinfection [6]. Given the rare nature of the condition, there are only a few studies or case reports dealing with this topic.

The purpose of this study was to review our institutional experience of IE in IVDUs, to identify predictive factors influencing the perioperative risk, and to establish perioperative management strategies with the aim of improving future clinical management and outcome.

## Methods

### Study design and patient cohort

From January 1993 to July 2013 a total of 451 patients underwent surgery for IE at University Hospital Heidelberg. Out of this cohort, 20 active IVDU patients were identified and included in the study. The study was approved by the Ethics Committee, Medical Faculty University of Heidelberg, application number S-198/2013. Patients were only included in the study if the endocarditis was considered to be “definite” according to the modified criteria proposed by Duke [7,8] and there was documented acknowledgement by the patient of active intravenous drug abuse (heroin) until the day of admission. Additionally, other drug use history was obtained including type of drug (marijuana, cocaine, amphetamines, ecstasy and methadone). Patient demographics, preoperative medical history, intra-operative and post-operative course were collected retrospectively from hospital medical records. Valve-related complications were documented according to the guidelines for reporting morbidity and mortality after cardiac valvular surgery [9]. Transesophageal echocardiography was performed in all survivors. Mortality was determined using the bureau of vital statistics database if such information was unobtainable from the medical records.

The primary endpoint in the study cohort was survival at 90 days after surgical treatment in our institution. The secondary endpoints were perioperative clinical characteristics and adverse events, which could have an impact on early postoperative mortality. All the patients completed a follow-up period of at least 90 days and were divided into two groups depending on 90-day survival. The demographic and perioperative variables of the 90-day survivors and non-survivors were compared to identify the predictors of 90-day overall mortality.

### Diagnostic methodology and indications for surgical treatment

The diagnosis of IE was made using a combination of clinical and laboratory findings and was confirmed by transesophageal echocardiography in all patients according to the modified Duke criteria. Mandatory preoperative studies included abdominal ultrasound or computer assisted tomography to rule out septic foci. Absolute indications for surgical treatment during the active phase of IE, according to our overall surgical principles at the University Hospital of Heidelberg, included the following: progressive cardiac failure, unmanageable infection despite antibiotic therapy, recurrent embolic events, acute renal failure and prosthetic valve endocarditis. Each patient in our cohort met at least one of these criteria.

### Surgical technique

All patients underwent surgery with cardiopulmonary bypass with mild hypothermia. Myocardial protection was achieved with antegrade cold crystalloid cardioplegia. Tricuspid valve surgical procedures were performed with occlusion of both caval veins while the heart was beating or fibrillating.

Our treatment policy for IE is radical debridement of infected tissue followed by generous irrigation with antibiotic solution. All infected or necrotic structures surrounding the valves were resected irrespective of whether the conduction system was damaged or large defects in the myocardial or fibrous tissue were created. The valvular annulus and adjacent structures were carefully examined with the aim of detecting extension of the infective process. Abscesses were resected as completely as possible so as not to leave any macroscopically infected tissue in situ. Large evacuated abscess cavities were filled with gentamycin-fibrin glue. Ventricular septal defects caused by extension of abscess cavities were closed with Dacron patches. Allografts were preferred for aortic root reconstruction in cases of annular destruction and periannular invasion. If there was a requirement to buttress the suture line of a prosthetic valve, autologous pericardium was the material of choice. Defects were subsequently closed with pericardial patches or if possible, by direct suturing. Generally, the use of foreign material was kept to a minimum.

If the valves had to be replaced, the type of valve implanted was generally determined by the preference of the surgeon. Prostheses, patch material, and sutures were soaked in gentamicin solution before implantation.

### Postoperative care

In addition to hemodynamic stabilization, an important goal of postoperative intensive therapy was to control the local and systemic infective process. In general, the antibiotic therapy was specifically directed against the infecting microorganisms. In the presence of methicillin-resistant staphylococci, vancomycin was used in combination with tobramycin or rifampicin. Methicillin-susceptible staphylococci were treated with a semisynthetic penicillinase-resistant penicillin and rifampicin. If no rapid disappearance of infective signs could be achieved, the therapy was changed to vancomycin alone or in combination. If no microorganisms could be isolated, broad spectrum antibiotic therapy with imipenem and vancomycin or teicoplanin was used. In each case, intravenous antibiotic therapy was maintained for 6 weeks postoperatively.

Post-operative transesophageal echocardiography was performed regularly to exclude recurrent vegetations or paravalvular leaks. These findings in combination with evidence of persisting infection would give a strong suspicion of ongoing or recurrent endocarditis and per se would justify re-operation.

### Statistical analysis

Data are presented as continuous or categorical variables. Continuous data were evaluated for normality using the one sample Kolmogorov-Smirnov test. As all continuous variables were normally distributed, they were analyzed with the Student *t*-test and expressed as the mean ± standard deviation. Pearson’s *χ*^2^ or Fisher exact tests were used for categorical data dependent on the minimum expected count in each cross tab. The categorical data are expressed as total numbers and percentages. The ANOVA with repeated measurements was applied for the comparison of survivors and non-survivors regarding perioperative inflammatory markers. Kaplan-Meier actuarial survival estimate was generated to analyse survival of the entire cohort. Multivariate logistic regression analysis was performed on univariate predictors for 90-days mortality with an entry criterion of p < 0.05. All data were analyzed using the Statistical Package for Social Sciences, version 21.0 (SPSS Inc., Chicago, Illinois).

## Results

### Perioperative outcome

The mean age of the entire population was 35 ± 7.7 years and 35% (n = 7) of the population were female. All patients in the study cohort were active IVDUs and had advanced heart failure with the mean NYHA stage of 3.4 ± 0.9. One patient from the cohort had previous aortic valve endocarditis and had undergone a previous aortic valve replacement. Further past medical history included active Hepatitis B (70.6%, n = 12), renal insufficiency (35%, n = 7), insulin dependent diabetes mellitus (5%, n = 1) and history of cerebrovascular event (5%, n = 1). The mean duration of i.v. drug abuse was 15.2 ± 10.6 years. The affected valves were aortic (20%, n = 4), mitral (10%, n = 2), tricuspid (40%, n = 8), combined aortic and mitral (10%, n = 2), combined aortic and tricuspid (5%, n = 1), combined mitral and tricuspid (10%, n = 2) and combined aortic, mitral and pulmonary (5%, n = 1) valves. Eighty per cent of the cohort (n = 16) had positive blood cultures and 85% (n = 17) had valve vegetations confirmed by transesophageal echocardiography. The mean size of the valve vegetations was 25 ± 9.4 mm. The surgery was performed through a median sternotomy using cardiopulmonary bypass (CPB) with a mean CPB time of 139 ± 78 min and cross clamp time of 92 ± 49 min. In cases of valve replacement, mechanical or biological prostheses were used in 55% (n = 11) and 40% (n = 8), respectively. In five percent of cases (n = 1) the valve was reconstructed. Moreover, one patient with severe aortic valve/root destruction had several abscess cavities including periannular invasion that were filled with gentamycin-fibrin glue and subsequently closed with pericardial patches. After that an allograft was anchored in the reconstructed aortic root. The other patient with extensive, infiltrative aortic and tricuspid valve endocarditis needed closure of a ventricular septal defect with a Dacron patch caused by extension of abscess cavities. Large parts of the walls of the atria and the right ventricle were destroyed by the infection, so that additional reconstruction with Dacron patches had to be performed. In 12 patients splenectomy was performed simultaneously for severe abscess formation, which was diagnosed preoperatively.

Thirty five per cent of cases (n = 7) were urgent, 60% (n = 12) were emergent and one case was performed as a salvage procedure. Perioperatively, patients received 1440 ± 991 mL of red blood cells, 410 ± 428 mL of fresh frozen plasma and 287 ± 330 mL of platelets. The mean operation duration was 236 ± 96 min. Postoperatively, all patients were transferred to the intensive care unit (mean stay 3.9 ± 3.1 days). Having achieved hemodynamic stability and after extubation, the patients were transferred to the high dependency unit (mean stay 12.6 ± 14 days) before they were discharged to normal surgical ward.

**Figure 1:**
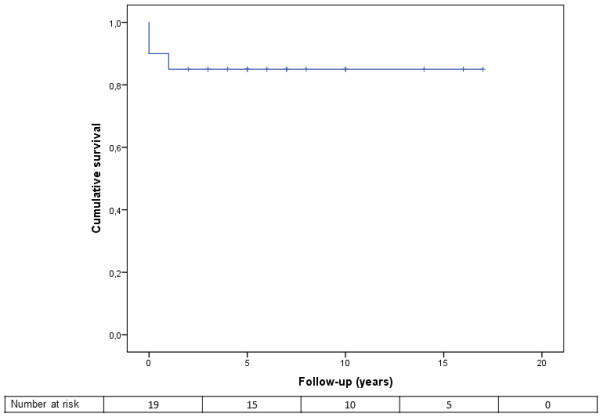
Kaplan-Meier survival estimate for IVDU patients with IE who underwent surgical treatment between January 1993 and July 2013.

The mean follow-up was 2504 ± 1842 days. The overall survival of the patient cohort was 90% at one year, and remained at 85% from 3 years to the end of follow-up (Figure 1). Seventeen patients survived until the end of follow-up, whereas 3 patients died at post-operative day 6, 8 and 591 days. The cause of death was severe sepsis in the first two cases and sudden cardiac death in the third. Eleven patients experienced recurrent IE during follow-up and were managed with i.v. antibiotics without the need for surgery. The most common postoperative adverse events were atrial fibrillation in 65% (n = 13), atrioventricular block in 25% (n = 5) with the need for permanent pacemaker implantation in 10% (n = 2), renal failure in 35% (n = 7), coagulation disorder (disseminated intravascular coagulation) in 20% (n = 4) and a cerebrovascular event (ischemic stroke) in 5% (n = 1). No myocardial infarction or deep sternal infections occurred postoperatively in the study cohort.

Staphylococcus aureus was the pathogen most commonly identified from blood cultures accounting for 40% (n = 8) of infections, followed by Enterococcus spec. 10% (n = 2), coagulase negative staphylococci 10% (n = 2) and streptococci 20% (n = 4). 20% (n = 4) of patients were culture-negative. The mean follow-up for the entire cohort was 2504 ± 1842 days. No patients were lost to follow-up.

### Univariate and multivariate predictors of early mortality

**Figure 2:**
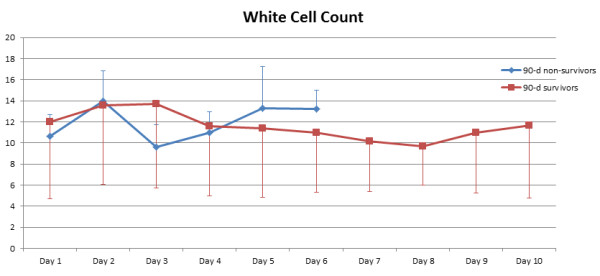
**Postoperative white cell count (WCC) course in 90-day survivors vs. 90-day non-survivors.** There are no statistically significant differences between the two groups (p = 1.000).

**Figure 3:**
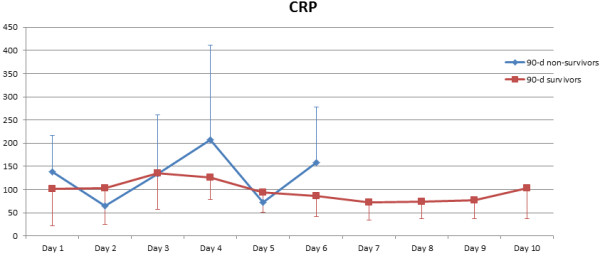
**Postoperative CRP course in 90-day survivors vs. 90-day non-survivors.** There are no statistically significant differences between the two groups (p = 0.275).

**Figure 4:**
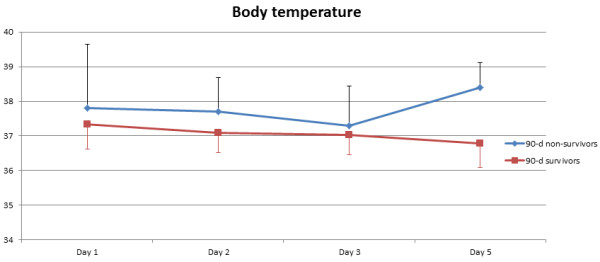
**Postoperative body temperature course in 90-day survivors vs. 90-day non-survivors.** Postoperative body temperature was significantly higher in the non-survivor group (p = 0.026) at all time points.

A subgroup analysis of 90-day survivors and non-survivors is presented in Tables 1 and 2. There were no statistically significant differences with respect to preoperative demographics and most clinical baseline characteristics including patients’ preoperative blood culture profile. However, non-survivors had significantly higher EuroSCORE II (p = 0.036), higher NYHA class (p = 0.005) and a significantly higher rate of HIV infection (p = 0.047). Also, non-survivors had a significantly longer operating time (p = 0.016) and higher RBC transfusion requirement (p = 0.020). Figures 2, 3, and 4 show the postoperative profile of white cell count (WCC), CRP and body temperature reflecting patients’ perioperative inflammatory status. In this respect, 90-day non-survivors had significantly higher body temperature over the first week postoperatively compared to 90-day survivors, while there were no significant differences regarding WCC and CRP levels. However, none of the univariate predictors reached statistical significance in multivariate logistic regression analysis.

**Table 1:** Patient preoperative demographics

	**90-d survivors**	**90-d non-survivors**	**p-value**
Age (yrs)	35.1 ± 7.7	34.5 ± 10.6	0.926
Female	6 (33.3%)	1 (50%)	0.639
BMI	23.8 ± 4.7	23.1 ± 0.42	0.851
Diabetes mellitus	1 (5.6%)	0	0.732
NYHA stage	3.28 ± 0.9	4.0 ± 0	0.005
Renal insufficiency	5 (33.3%)	1 (50%)	0.199
Hepatitis B	11 (73.3%)	1 (50%)	0.496
HIV	1 (5.6%)	1 (50%)	0.047
Previous CVA	1 (5.6%)	0	0.732
Vegetation	15 (93.8%)	2 (100%)	0.716
Previous endocarditis	1 (5.6%)	0	0.732
GFR (mL/min/1.73 m^2^)	89.2 ± 43.2	82.5 ± 82.7	0.848
Creatinine (mg/dL)	1.9 ± 2.4	2.3 ± 2.1	0.855
Urea (mg/dL)	73.1 ± 60.4	73.5 ± 51.6	0.992
GGT (U/L)	88.4 ± 69.7	18.5 ± 0.7	0.186
ALT (U/L)	57.9 ± 43.6	10.5 ± 0.7	0.154
AST (U/L)	107.6 ± 121.3	258.5 ± 334.5	0.182
Bilirubin (mg/dL)	1.62 ± 3.05	0.6 ± 0.35	0.636
*Patient’s preoperative blood culture profile*			0.504
Negative (marantic endocarditis)	4 (22.2%)	0	
Staphylococcus aureus	6 (33.3%)	2 (100%)	
Enterococcus	2 (11.1%)	0	
Coagulase negative Staphylococcus	2 (11.1%)	0	
Streptococcus species	4 (22.2%)	0	
EuroSCORE II	6.89 ± 3.35	11.04 ± 3.35	0.036

*BMI*, body mass index; *NYHA*, New York Heart Association; *HIV*, human immunodeficiency virus; *CVA*, cerebrovascular event; *GFR*, glomerular filtration rate; *GGT*, gamma glutamyl transpeptidase; *ALT*, alanine aminotransferase; *AST*, Aspartate transaminase.

**Table 2:** Intraoperative data and postoperative outcomes

	**90-d survivors**	**90-d non-survivors**	**p-value**
Vegetation size (mm)	23.5 ± 8.7	35 ± 7.1	0.099
Bypass time (min)	130.4 ± 70.9	219.5 ± 120.9	0.126
Cross clamp time (min)	86.7 ± 44.4	136 ± 84.9	0.189
Operation duration (min)	219 ± 78	385 ± 148	0.016
*Type of valve surgery*			0.189
Mechanical prosthesis	11 (61.1%)	0	
Biological prosthesis	6 (33.3%)	2 (100%)	
Reconstruction	1 (5.6%)	0	
*Site of valve surgery*			0.113
Aortic	4 (22.2%)	0	
Mitral	2 (11.1%)	0	
Tricuspid	7 (38.9%)	1 (50%)	
Aortic and mitral	2 (11.1%)	0	
Aortic and tricuspid	1 (5.6%)	0	
Mitral and tricuspid	2 (11.1%)	0	
Aortic, mitral and pulmonary	0	1 (50%)	
*Multivalve surgery*	5 (27.8%)	1 (50%)	0.521
RBC (mL)	1267 ± 883	2900 ± 141	0.020
FFP (mL)	433 ± 441	200 ± 283	0.479
Platelets (mL)	267 ± 305	440 ± 622	0.503
Atrial fibrillation	11 (61.1%)	2 (100%)	0.521
AV-block	4 (23.5%)	1 (50%)	0.468
Permanent pacemaker	2 (11.8%)	0	1.000
Renal failure	5 (27.8%)	2 (100%)	0.111
Coagulation disorder	3 (17.6%)	1 (50%)	0.386
Reopening for bleeding	0	0	
CVA	0	0	

*RBC*, red blood cells; *FFP*, fresh frozen plasma; *CVA*, cerebrovascular accident.

## Discussion

The management of IE in active IVDUs remains challenging and has a high operative mortality. Often the surgeon faces a very sick patient with septic complications, which need to be treated surgically as soon as possible after failed antibiotic therapy. This report summarizes our institutional experience of this high-risk group treated surgically over a 20-year period. We critically analysed our surgical treatment of IVDUs, in particular the causative pathogens with the aim of identifying methods, which can improve the outcome of this sub-group of patients.

Ninety percent of our patients survived to hospital discharge, a survival rate that is comparable with reports of other institutions [10-12]. The fact that 35% of the cases were urgent, 60% were emergent and one case was a salvage procedure reflects the severe stage of the disease in our cohort. Nevertheless, we were able to achieve a long-term survival of 85%.

Staphylococcus aureus was the most common pathogen isolated in our patient group but was not associated with significantly decreased 90-day survival when compared with the other pathogens. In contrast, other investigators have reported that Staphylococcus aureus - associated IE was identified as the most common cause of IE and is an independent predictor of mortality [13,14]. For this reason, we changed the antibiotic therapy in Staphylococcal IE to vancomycin and rifampicin if the preoperative antibiotic regime was ineffective post-operatively. Our study data showed that patients receiving concentrations of ≥10 mg/L vancomycin are more likely to become afebrile and most of them have a normal white blood cell count within 72 hours.

Staphylococci are prone to cause abscess formation and early extracardiac organ manifestations. Fowler reported that infection with Staphylococcus aureus was associated with decreased survival compared to non – Staphylococcus aureus infection [13]. This suggests surgical treatment should be performed earlier when the patient is in a less advanced state of systemic infection. In the current study, two patients died 6 and 8 days after surgery, whereas one patient died after 591 days. The cause of death was severe sepsis in the first two cases and sudden cardiac death in the third. An important finding was that these patients also had significantly longer operation time and higher need for red blood cell transfusions when compared to survivors. However, in most cases we cannot influence the intraoperative course as it is primarily determined by the nature of the disease.

The patients who died before post-operative day 30 suffered from severe bacteremia caused by Staphylococcus aureus and when admitted to our hospital, were mechanically ventilated and inotrope-dependent. A previous report from Van Duin has already demonstrated 1-year mortality of 40% in patients with Staphylococcus aureus bacteremia from any source with an attributable mortality ranging between 8% and 16% [15]. Unfortunately, we cannot provide additional information for the patient who died after 591 days, because he was found dead at home and no post mortem report was available.

In our experience, the preoperative NYHA class was a significant predictor of early mortality. All patients who died in the first 90 days after operation were in NYHA class IV. This could be a strong argument to operate on IVDUs with IE expeditiously, before the onset of acute heart failure. A previous report also demonstrated similar results with patients arriving in cardiogenic shock in which it was concluded that immediate surgery was the only viable treatment option [16].

In this study there were no reoperations and no recurrent infection in spite of all patients being in the active phase of endocarditis. In our opinion this demonstrates our success with the radical approach in our institution. However, radical surgery can compromise structures like the cardiac conduction system; it resulted in the onset of complete AV block postoperatively in two patients necessitating pacemaker implantation. However, we believe that the deleterious effects of radical surgery are offset by the benefits associated with the effective removal of infected tissue.

All of our patients were high frequency heroin abusers. Heroin abuse is associated with bacteremia and endothelial injury, simultaneously depressing respiratory function and increasing pulmonary artery pressure significantly. The same applies to cocaine and methamphetamine injections, which are associated with bacteremia, turbulent flows in the heart and increased systemic afterload. These combined effects can potentiate mitral and aortic valve insufficiency and alter local blood flow in the valve region, which may increase the risk of IE [17].

The surgeon who is considering placing a bioprosthetic valve in an IVDU patient with IE has to make challenging decisions regarding long-term durability with the potential need for reoperation and anticipated patient compliance with anticoagulation treatment. More than the half of our patients received mechanical prostheses primarily because they expressed reservations about receiving a bioprosthesis because of durability concerns and by persuading their surgeons that they would be medically compliant and stop i.v. drug abuse. Grover [18] found no differences in the recurrence of IE between recipients of mechanical and bioprosthetic valves. Bauernschmitt [19] reported encouraging results with the use of mechanical prostheses in IE. The findings of these two studies, in combination with those of the current study, support the use of a mechanical prosthesis in IE.

In the current study, the operative risk associated with multiple valve involvement did not exceed the risk of single-valve disease, irrespective of affected valve type, and was not accompanied by a significant increased 90-day mortality. Nevertheless, previously it has been shown that multivalve endocarditis is a rare clinical entity with a substantially elevated risk of morbidity and mortality [20].

Some authors have identified coinfection with HIV and the degree of immunosuppression as a risk factor of mortality in IVDU patients with IE, particularly in patients with CD4 cell counts <200/mm^3^[10,11,21]. In our population, 10% of the patients were HIV infected. We did not find any significant differences in the mortality rate according to the HIV infection serostatus, which may have been attributable to the low number of affected patients.

Other studies have identified the presence of valvular vegetation as a risk factor for complications [22-25]. Mitral valve endocarditis with large staphylococcal vegetations in particular has been shown to be an independent predictor of stroke [25], and this complication occurred preoperatively in one patient in the current study. Generally, these vegetations are associated with a high concentration of pathogens, are rarely responsive to antibiotic treatment and represent a source of embolic events [12]. Moreover, in some studies it has been demonstrated that vegetation size is predictive of outcome [22-24] but this conclusion is not supported by another study which failed to show an association between prognosis and vegetation characteristics [12,26]. Transesophageal echocardiography is effective in determining the location and size of vegetations, but in right-sided endocarditis, offers no advantages over transthoracic imaging [26]. Nevertheless, transesophageal echocardiography remains a mandatory requirement and represents the gold standard in IE evaluation.

### Limitations

The main limitation of this study is its retrospective single-arm, non-randomized design involving a limited number of patients from a single institution leading to a low statistical power. Given the rare nature of the condition, it was not possible to get a large number of patients and so, a prospective multi-centre long-term study should be conducted to further characterize IE in IVDUs in a larger patient sample. In spite of these limitations, we were able to include consecutive patients and were able to retrieve complete data records from the majority. Theoretically, it would have been desirable to compare outcomes of study patients with a medically treated control group although this was deemed to be ethically infeasible; all patients were deemed to be too sick for further antibiotic therapy and thus were treated surgically. The onset of IE did not necessarily occur at the time of the initiation antibiotic therapy. As most patients were admitted by external hospitals, and many of them were treated by their primary physicians, the exact onset of the endocarditis could not be defined in each patient.

## Conclusions

Despite progress in antibiotic therapy and cardiac surgical management, surgery of IE in active IVDUs remains demanding, because it entails high-risk surgical treatment and targeted postoperative intensive care. An early diagnosis is a critical determinant of final outcome. In summary, the key to long-term success in IE is early and radical surgical debridement of the infected tissue followed by an aggressive postoperative antibiotic therapy.

## Abbreviations

IE: Infective endocarditis; IVDUs: Intravenous drug users; HACEK: (Haemophilus species, Actinobacillus actinomycetemcomitans, Cardiobacterium hominis, Eikenella corrodens, Kingella kingae); CPB: Cardiopulmonary bypass; NYHA: New York Heart Association; RBC: Red blood cells; WCC: White blood cells; CRP: C reactive protein.

## Competing interests

The authors report no competing interests.

## Authors’ contributions

AW and TB designed the study; AW, TB and AS performed the statistical analysis and drafted the manuscript; NC, BS, GV and TB were involved in collecting data and drafting the manuscript; AP, MK, CB and GS helped to draft the manuscript; GS, CB and AS gave critical comments on the results. All authors read and approved the final manuscript.
